# Classification of healthy and diseased retina using SD-OCT imaging and Random Forest algorithm

**DOI:** 10.1371/journal.pone.0198281

**Published:** 2018-06-04

**Authors:** Md Akter Hussain, Alauddin Bhuiyan, Chi D. Luu, R. Theodore Smith, Robyn H. Guymer, Hiroshi Ishikawa, Joel S. Schuman, Kotagiri Ramamohanarao

**Affiliations:** 1 Computing and Information Systems, The University of Melbourne, Melbourne, Australia; 2 iHealthScreen Inc., Queens, New York, United States of America; 3 Centre for Eye Research Australia, Royal Victorian Eye and Ear Hospital, Department of Surgery (Ophthalmology), The University of Melbourne, Melbourne, Australia; 4 Icahn School of Medicine at Mount Sinai, New York, New York, United States of America; 5 New York University School of Medicine, New York, New York, United States of America; Massachusetts Eye & Ear Infirmary, Harvard Medical School, UNITED STATES

## Abstract

In this paper, we propose a novel classification model for automatically identifying individuals with age-related macular degeneration (AMD) or Diabetic Macular Edema (DME) using retinal features from Spectral Domain Optical Coherence Tomography (SD-OCT) images. Our classification method uses retinal features such as the thickness of the retina and the thickness of the individual retinal layers, and the volume of the pathologies such as drusen and hyper-reflective intra-retinal spots. We extract automatically, ten clinically important retinal features by segmenting individual SD-OCT images for classification purposes. The effectiveness of the extracted features is evaluated using several classification methods such as Random Forrest on 251 (59 normal, 177 AMD and 15 DME) subjects. We have performed 15-fold cross-validation tests for three phenotypes; DME, AMD and normal cases using these data sets and achieved accuracy of more than 95% on each data set with the classification method using Random Forrest. When we trained the system as a two-class problem of normal and eye with pathology, using the Random Forrest classifier, we obtained an accuracy of more than 96%. The area under the receiver operating characteristic curve (AUC) finds a value of 0.99 for each dataset. We have also shown the performance of four state-of-the-methods for classification the eye participants and found that our proposed method showed the best accuracy.

## Introduction

Eye diseases such as Age-related Macular Degeneration (AMD) and Diabetic Macular Edema (DME) are amongst the most common causes of vision loss in our communities. The number of people with AMD is expected to increase by ≈1.5 fold over the next ten years due to an increase in aging population [1]. Similarly cases of DME are expected to grow exponentially affecting over 300 million people worldwide in the next few years [2, 3]. In this paper, we have proposed an automatic classification method for people with AMD, DME, and people with normal retinae using Random Forest (RF), a highly robust and efficient machine-learning algorithm. The classification method might be able to be used to determine the severity level of disease based upon their risk of progression and potentially serve as a prediction tool. A total of ten features, based upon current clinical knowledge, have been extracted automatically from the spectral domain optical coherence tomography (SD-OCT) retinal images of patients. We have tested several machine learning algorithms such as support vector machine (SVM), Decision Tree, etc. Among them, RF has shown the best performance (more than 97% accuracy) overall.

**Fig 1:**
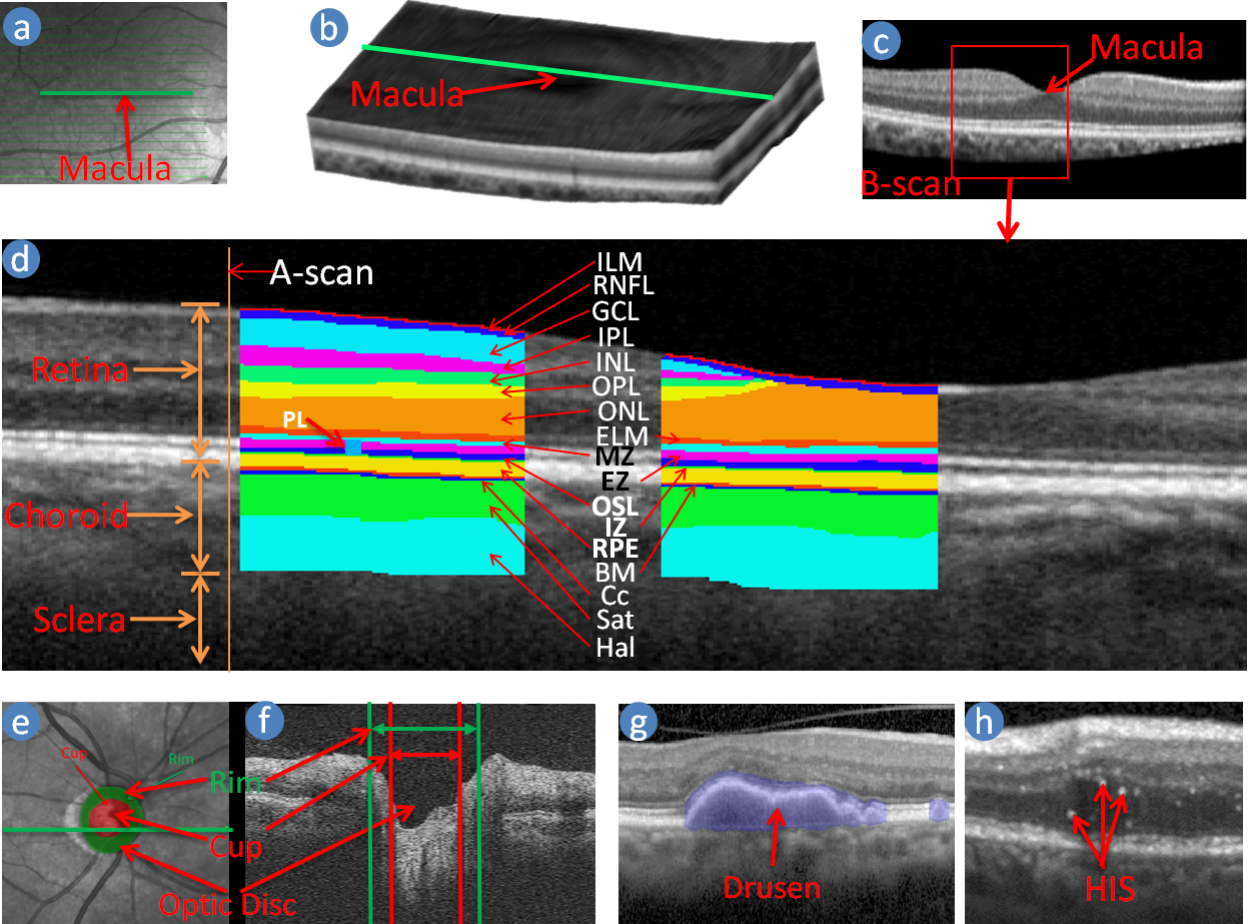
Macula centred retinal image. (a) Near infra-red image; (b) Volume or 3D reconstruction of the retina from OCT scans; (c) A B-scan image (cross-section of the retina through the green line in Fig a and b), (d) Retinal Layers are delineated in a B-scan image, (e) and (f) show Optic disc centred enface and SD-OCT B-scan respectively, (g) and (h) show drusen and hyper-reflective intra-retinal spots (HIS) in SD-OCT B-scan respectively.

SD-OCT technology is a non-invasive method of obtaining images of the retina in 3D where more information of the retina is available than is commonly the case when using 2D imaging such as colour fundus photography (CFP) [4]. CFP cannot always identify signs of disease within the retina such as cysts [2] nor provide any information on subtle changes in the retina thickness or morphological information. SD-OCT uses the back-scattered light and interferometer that uses a low coherence light source to image various tissue layers [5]. The laser or infra-red light penetrates through the depth of the retinal tissue along a point and reads data regarding the backscattered light intensity and coherence, creating what is known as an axial scan (A-scan) shown in Fig 1(d) [6]. These single A-scans can be assembled linearly across the tissue (green line in Fig 1(a))—making one cross-sectional image which is known as a B-scan (Fig 1(c)), and a pool of parallel B-scans form a 3D structure of the retina and choroid (Fig 1(b)). Anatomically the retinal tissues can be classified into ten layers with the choroidal structure beneath the retina as shown in Fig 1(d) [7]. Different layers of the retina, the components of the choroid and the pathologies such as drusen and Hyper-reflective Intra-retinal Spots (HIS), are observable in the OCT image as shown in Fig 1(g) and 1(h) using the variation of the intensities and coherence due to reflective nature of the tissues and their thicknesses, vascular structures, pathologies, etc. [1, 5, 8, 9] Fig 1(d) shows a B-scan delineating the ten retinal layers and choroid components [7]. The ten layers of the retina are: 1) Inner Limiting Membrane (ILM); 2) Retinal Nerve Fibre Layer (RNFL); 3) Ganglion Cell Layer (GCL); 4) Inner Plexiform Layer (IPL); 5) Inner Nuclear Layer (INL); 6) Outer Plexiform Layer (OPL); 7) Outer Nuclear Layer (ONL); 8) External Limiting Membrane (ELM); 9) Photoreceptor Layer (PL); 10) Retinal pigment epithelium (RPE). The PL is divided into three segments, and they are 1) Myoid Zone (MZ); 2) Ellipsoid Zone (EZ); 3) Outer Segment Layer (OSL). The boundary between the RPE and the choroid is Bruch’s membrane (BM) [7, 10]. The two major components of the choroid are the choroidal vessel and stroma [11]. The choroidal vessel is sub-divided into three layers: the choriocapillaris (Cc), Sattler’s (Sat) and Haller’s (Hal) layers. Most of the time, the boundaries between the RPE, Bruch’s membrane, and choriocapillaris, known as RBC complex, are indistinguishable due to the signal attenuation of the image [11, 12]. The retinal thickness is defined by the boundary of ILM-RNFL and RBC. Much of the previous work on OCT image analysis has focused on the problem of retinal layer segmentation, which is a necessary step for retinal and its constitutive layer thickness measurements [11, 12].

There has been some work on the automatic segmentation of the retinal layers, but only a few methods are available for the classification of the SD-OCT volumes [13–15]. Among those classification methods, most of them are binary classifier that is, classified into diseased or normal cases, not specific diseases such as AMD and DME. Almost all of those methods designed the classification method using the texture information of the SD-OCT images. Fraccaro et al. [15] developed a classification method using retinal pathology information, but they were manually extracted features. To the best of our knowledge, there is no classification method based on automatically segmented the retinal structure and pathological information. We discuss the background work on the classification method in section Literature Review.

In this paper, we propose a classification method of AMD, DME and normal individuals. The system has also been tested for the binary classification case that has also shown excellent performance. There are a total of ten retinal features extracted from the SD-OCT images, and all of these are considered clinically important features based on the changes of the retinal structure and pathology due to AMD and DME. The retinal layers are automatically segmented using our method [16]. We have proposed a quantification method for pathology with the help of segmented layer. The features constructed with two parameters each from the retinal thickness, the complex of ELM to RPE layer, and the RPE layer; two parameters from the boundary curviness of the retinal layers (OPL-ONL and MZ-EZ), and two parameters for the volume of the RPE detachment (drusen) and hyper-reflective intra-retinal spots. Several machine-learning approaches have been used to test the performance of the feature selection as well as a comparison between them. Evaluation is performed on seven datasets from four different sources including two public dataset [17, 18] (see section Dataset and Experiment Setup and Result) with fifteen-fold cross validation such that each test includes one case of each for the data set containing 45 individuals. The results show high accuracy on all seven datasets and higher than the original work on the public dataset [17].

Contributions of the paper are as follows.

1. Automatic classification of SD-OCT volumes of patients into AMD, DME, and normal individuals.
2. Automatic feature extraction from the SD-OCT volumes that are related to the changes of the retinal structure due to AMD and DME (such as thickness of the retina and retinal layers, drusen).

## Literature review

There are few works available that classify patients into various diseases or normal based on the retinal SD-OCT images. Most of these works use a binary classifying system into diseased or normal. The features for classification purposes are mostly on texture information of the image and are created using Local Binary Pattern (LBP), a histogram of oriented gradient and other texture analysis. These features are filtered using principle component analysis (PCA), Bag-of-word, and k-means cluster, etc. The disadvantage of the texture information is that it is more susceptible to noises and device oriented due to different intensity variation among them. On the other hand, retinal structure information does not depend on the device and is less susceptible to noise. That is why the classification method based on retinal structure information is more reliable than the texture-based classification. Fraccaro et al. [15] showed that Random Forest algorithm had superior performance when compared to One-rule, Decision Tree, Logistic Regression, AdaBoost, and Support vector machine for the classification of disease. A brief summary of the classification methods used in eye disease is as follows.

Liu et al. [13] proposed a method for macular pathology detection in OCT images using Local Binary Patterns (LBP) and gradient information as attributes. The method starts by aligning and flattening the images, and then a 3-level multi-scale spatial pyramid is created. From every level of the pyramid, edge and LBP histograms are extracted in each block. The obtained histograms are used to form a global descriptor. The principle component analysis is used to reduce the dimension of the global descriptor. Finally, a two-class, non-linear support vector machine is used to train the system and classify the SD-OCT volume into normal macula and three macular pathologies (macular hole, macular edema, and AMD). They used 193 volumes from 136 subjects for training the system and 58 volumes from 37 subjects for the testing the system. The cross-validation area under the receiver operating curve (AUC) on the development dataset was 0.976, 0.931, 0.939, and 0.938, and the AUC result on the holdout testing set was 0.978, 0.969, 0.941, and 0.975, for identifying normal macula, Macular Hole (MH), Macular Edema (ME), and AMD, respectively.

Albarrak et al. [14] proposed a decomposition based approach for classifying the participants into normal or AMD cases. After de-noising the image, they flatten the image and cropped an interest of volume for extracting 192 histogram bins as features using a normal LBP histogram and a Histogram of Oriented Gradients (HOG) for LBP-TOP on XY, XZ, and YZ planes. A Bayesian network classifier was then used to categorise the subjects. The proposed technique was evaluated using ten-fold cross validation to 140 volumetric OCT images and demonstrated a promising performance with the best AUC value of 94.4%.

Srinivasan et al. [17] proposed a classification method to distinguish DME, AMD, and normal SD-OCT volumes. After de-noising, the image using the sparsity-based block matching and 3D-filtering, flatten the image based on estimated RPE layer position and crop the region of interest for extracting features for the classifier. The features extracted for each slice of a volume using HOG and a linear Support Vector Machines (SVM) is used for classification. On a dataset of 45 patients equally subdivided into the three aforementioned classes, this method leads to a correct classification rate of 95.56% for DME, AMD and normal patients.

Venhuizen et al. [19] also proposed a method for OCT images classification into AMD and normal participants using the Bag-of-Words (BoW) models. The method selected the key points in each B-scan from where 9 × 9 patches are extracted around each key point. The dimension of the patches is reduced by using the PCA and created a codebook using k-means clustering. The obtained codebook from the training is used to represent each OCT volume as a feature vector occurrence histogram. Finally, Random Forest (RF) with a maximum of 100 trees is used for the classifier. The method achieved an AUC of 0.984 with a dataset of 384 (269 AMD, 115 control) OCT volumes.

Fraccaro et al. [15] proposed a method for AMD and normal participant classification from SD-OCT images using various machine learning approaches and showed Random Forest perform best compare to others. They have used manual segmentation of the druse and other pathologies from 912 volumes of 487 patients. They tested Decision Tree, Logistic Regression, AdaBoost, Support vector machine and Random Forest algorithms. Regarding AUC, random forests, logistic regression and AdaBoost showed a mean performance of (0.92), followed by SVM and decision trees (0.90).

Lemaitre et al. [2] proposed a method for automatic classification of patients into DME and normal subjects from SD-OCT volumes. Their method was based on LBP features to describe the texture of OCT images and dictionary learning using the BoW models. The images were divided into local patches and extracted a dense set of LBP descriptors. They had extracted 3D-LBP features from the entire OCT volume and used Random Forest classifier. They had used two datasets from two different sources and consisted 32 (16 DME and 16 Normal) volumes from Singapore Eye Research Institute (SERI) and 30 (15 DME and 15 Normal) from Srinivasan et al. [17]. They achieved approximately 87% sensitivity and 75% specificity over the two datasets.

Sidibe et al. [20] proposed a classification model for DME patients by modelling the appearance of normal OCT images with a Gaussian Mixture Model (GMM) and detecting abnormal OCT images as outliers. The classification of an OCT volume was based on the number of detected outliers. They used the same dataset of Lemaitre et al. [2] and showed better output than Lemaitre et al. [2] and Venhuizen et al. [19]. They achieved a sensitivity and a specificity of 100% and 80% on the Duke dataset [17].

In conclusion, the above methods do not consider structural features of the retina such as reduction in layer thickness, drusen characteristics, etc. whereas our method is based upon features that are considered clinically important.

## The proposed method

**Fig 2:**
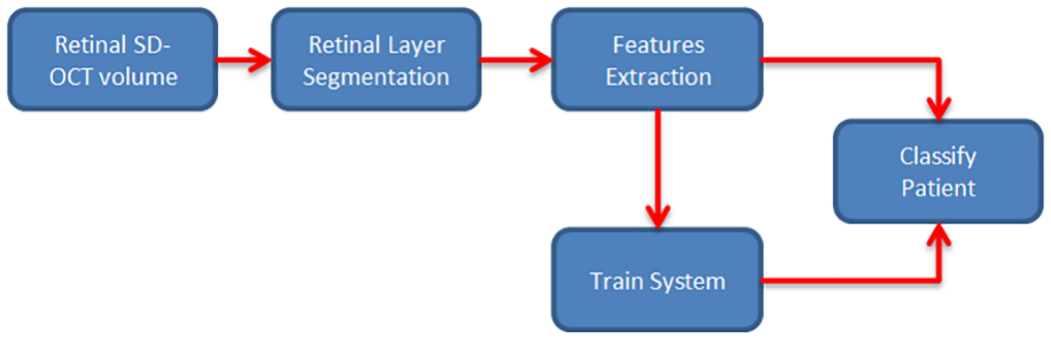
The basic flow diagram. The flow diagram of the proposed classification method.

The methodology of classification of the diseased eyes is formulated as a standard classification procedure as shown in Fig 2. Since we use the segmentation method proposed in [11] to extract retinal layer boundaries there is no need for pre-processing or noise reduction of the input images. After the segmentation, ten retinal features are extracted as described in section **The Feature Extraction Process**. The difference between boundaries of a layer along the A-scan is considered as the thickness of the layers and retina. The pathologies such as drusen and Hyper-reflective Intra-retinal Spots (HIS) are identified using position and intensity profiling of the image. Layer segmentation is used to identify the position and hence the type of pathology. For example, drusen are identified using the non-uniformity of thickness of the RPE layer and by simply counting the number of pixels in the drusen, the volume of the drusen is derived. Training and testing are two steps of the machine learning algorithm where training is used to create the classification model, and testing is used to evaluate the performance of the model. In the next two subsections, a brief description of automatically segmented retinal layers and extracting the features are discussed.

### Segmentation of the boundaries of the retinal layers

In general, the borders of the tissue layers of the retina are smooth horizontal lines in normal persons. This property can be exploited for segmentation of layers by mapping the problem as the shortest path graph problem. The graph nodes are formed using the edge pixels found with Canny Edge Detection Algorithm. Edge weights between the nodes are computed using features such as distance between the nodes, the slope similarity to a reference line and node’s non-associativity (pixels not satisfying associated layer property) to the layer [11]. Once such a graph is constructed, the layers boundary can be determined by computing the shortest path between the start A-scan and end A-scan of a given B-scan image.

**Fig 3:**
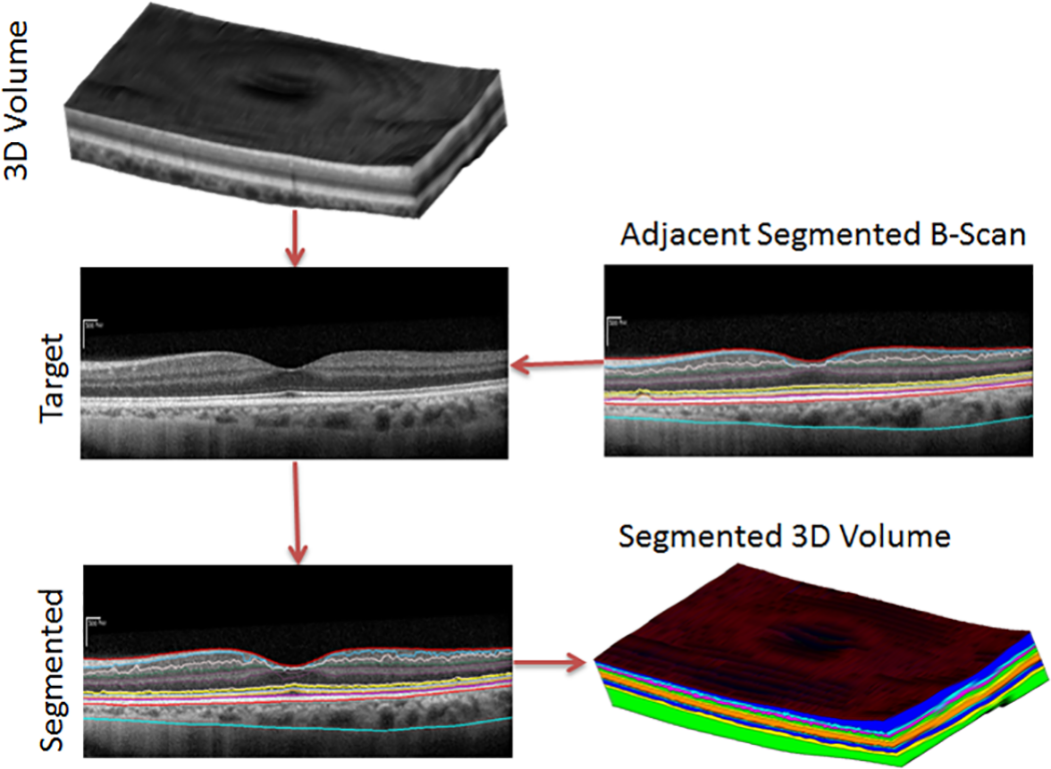
The basic flow diagram of the segmentation algorithm. The basic flow diagram of our proposed 3D segmentation.

**Fig 4:**
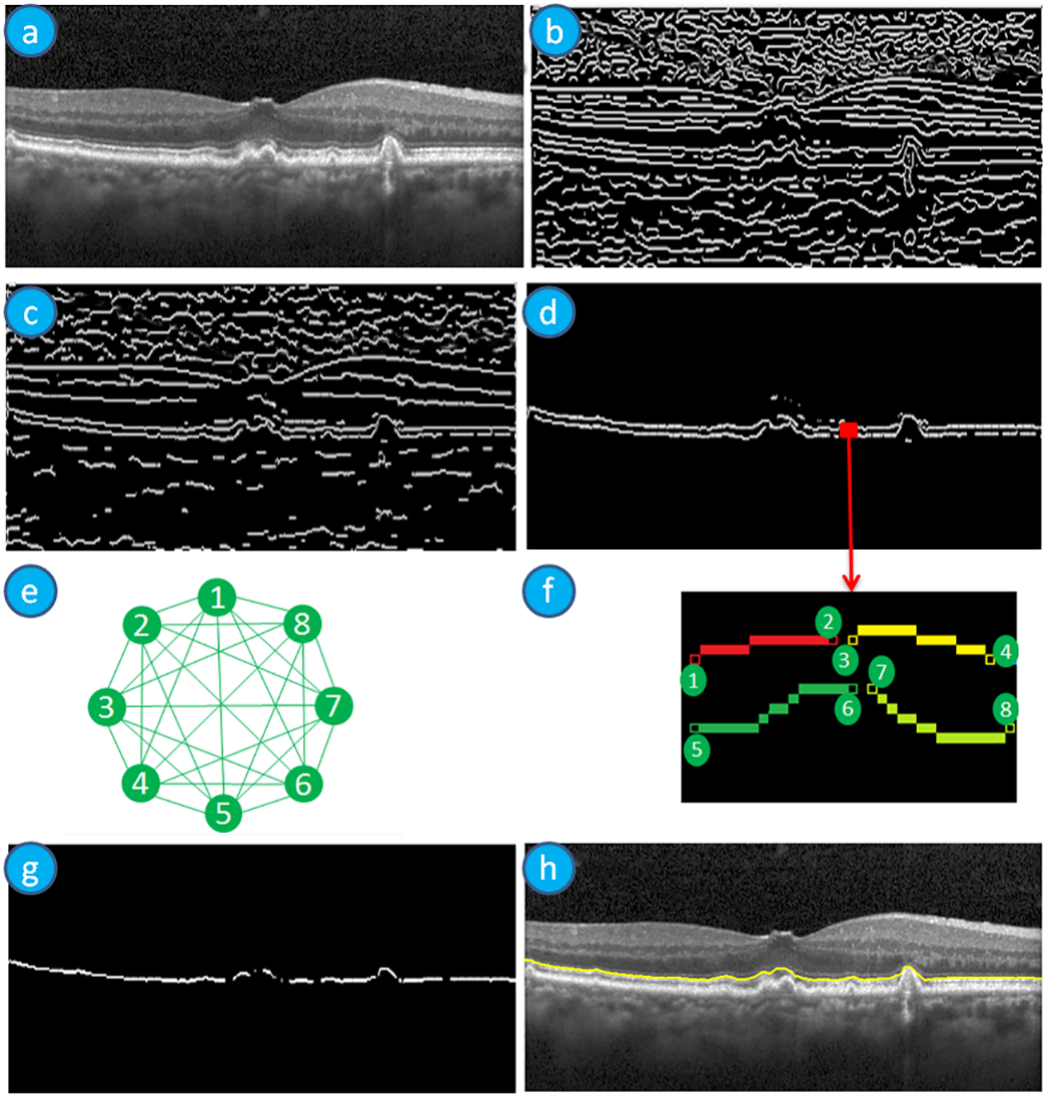
MZ-EZ boundary detection steps of SD-OCT B-Scan Image. (a) SD-OCT B-Scan image; (b) Image after applying Canny edge detection highlighting edge pixels; (c) Image with highlighted edge pixels having positive intensity gradient; (d) Image with the candidate pixels in the ROI which is defined by the upper and lower boundaries depending on the target boundary; (e) The partial graph of the full connected graph representation of the boundary detection problem; (f) A magnified image of the red region of (d), each colour represents a different pixels-group and black circles represent the end pixels which are the nodes of the graph; (g) Image with highlighted pixel-groups lying on the shortest path of the graph; (h) The MZ-EZ Boundary (yellow line) is detected after fitting a curve.

Also, the tissues of the retina are continuous in adjacent B-scans when the distance between the adjacent scans are very close. Therefore, we expect very small changes of a boundary from one B-scan to the next. This information helps to correct boundaries by using neighbourhood information. Therefore, we employ 3D segmentation in our method to overcome 2D segmentation failures due to noise or tissue structure or pathologies. Our method first detects the boundaries sequentially in the order of high contrast and the maximum gradient intensity to low contrast and minimum gradient intensity of the boundaries. This approach helps to detect the low contrast boundaries in a small region of interest (ROI) since we define ROI using the already detected boundaries and adjacent B-scans. The reduction of the ROI helps to improve the accuracy and efficiency of the detection even in the presence of pathologies. The sequence of the detection of boundaries is 1) ILM-RNFL, 2) RBC, 3) MZ-EZ 4) IZ-RPE 5) OPL-ONL, 6) ONL-ELM, 7) EZ-OSL, 8) ELM-MZ, 9) INL-OPL, 10) IPL-INL, 11) RNFL-GCL, and 12) GCL-IPL. The layer-specific region of interest selection and approximation of three prominent reference layers makes our method efficient, accurate and robust. Fig 3 shows a basic flow diagram of our proposed 3D segmentation. A brief description of the segmentation of the MZ-EZ boundaries is explained in Fig 4. In Fig 4, a target SD-OCT B-scan is shown in (a); edge pixels after applying Canny edge detection highlighting in (b); since MZ-EZ boundary have positive intensity gradient, we select only those edge pixels with positive gradient as shown in (c); the candidate pixels in the region of interest (ROI) which is defined by the upper and lower boundaries is shown in (d); A magnified image of the red region of (d) is shown in (f), each colour represents a different pixels-group and black circles represent the end pixels which are the nodes of the graph; The graph representing edge pixels-groups of (f) is shown in (e). To make it clear, (e) shows only a partial graph and it does not show edges coming into the graph and leaving the graph to its neighbouring edge pixel-groups; pixel-groups lying on the shortest path of the graph is shown in (g); reconstructed MZ-EZ Boundary (yellow line) is detected after fitting a curve as shown in (h).

### The feature extraction process

Ophthalmologists have defined a set of anatomical signs from the changes of the retinal structural information that are seen in retinal diseases such as AMD and DME [21–23]. The signs include abnormality of the retina and its layer thickness and reflectivity [21, 22] such as where the OSL thickness was reported to be reduced significantly in early AMD patients [24]. Ophthalmologists have also defined presence of pathologies such as drusen and RPE detachments for AMD and intra retinal cysts for DME [22]. As a consequence, the proposed method has used these signs of DME and AMD as features for the classification method. A total of ten features are extracted via a process as follows.

#### Feature 1: Volume of the HIS

**Fig 5:**
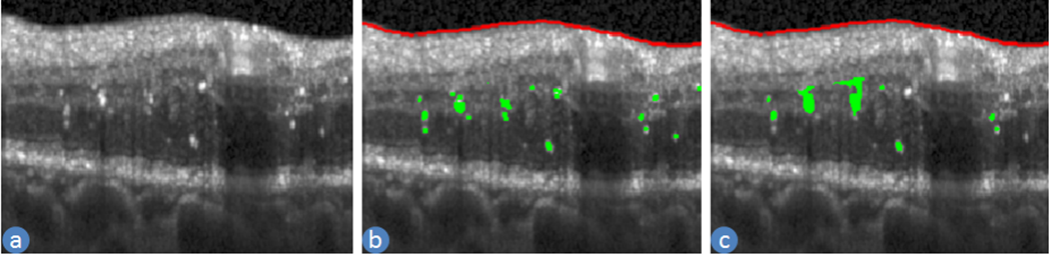
An example of HIS segmentation. (a) an SD-OCT B-scan (b) manual ILM-RNFL boundary (red line) and HIS (green colour) (c) automatically detected ILM-RNFL boundary (red line) and HIS (green colour).

The presence of the hyper-reflective intra-retinal spots (HIS) in the retinal SD-OCT volumes present in diabetic eyes, can occur even when clinical retinopathy is undetectable clinically [23]. For this reason, we have chosen the volume of the HIS as a feature. It is characterised by the brighter intensity and located in the inner retina mostly in INL to ONL layer as shown in Fig 1(h). Since we have segmented the layers, we searched for HIS only in these layers. In general, these layers are darker than RNFL and RPE layers except for the HIS pixels. So the pixels, which are located in INL to ONL layer and have an intensity value more than the mean intensity of the RNFL and RPE layers, are defined as HIS pixels. The total number of pixels multiplied by the resolution of the image is considered as the volume of the HIS. Fig 5 shows an example of the automatic segmentation of HIS by our proposed method.

#### Feature 2: Volume of the drusen

**Fig 6:**
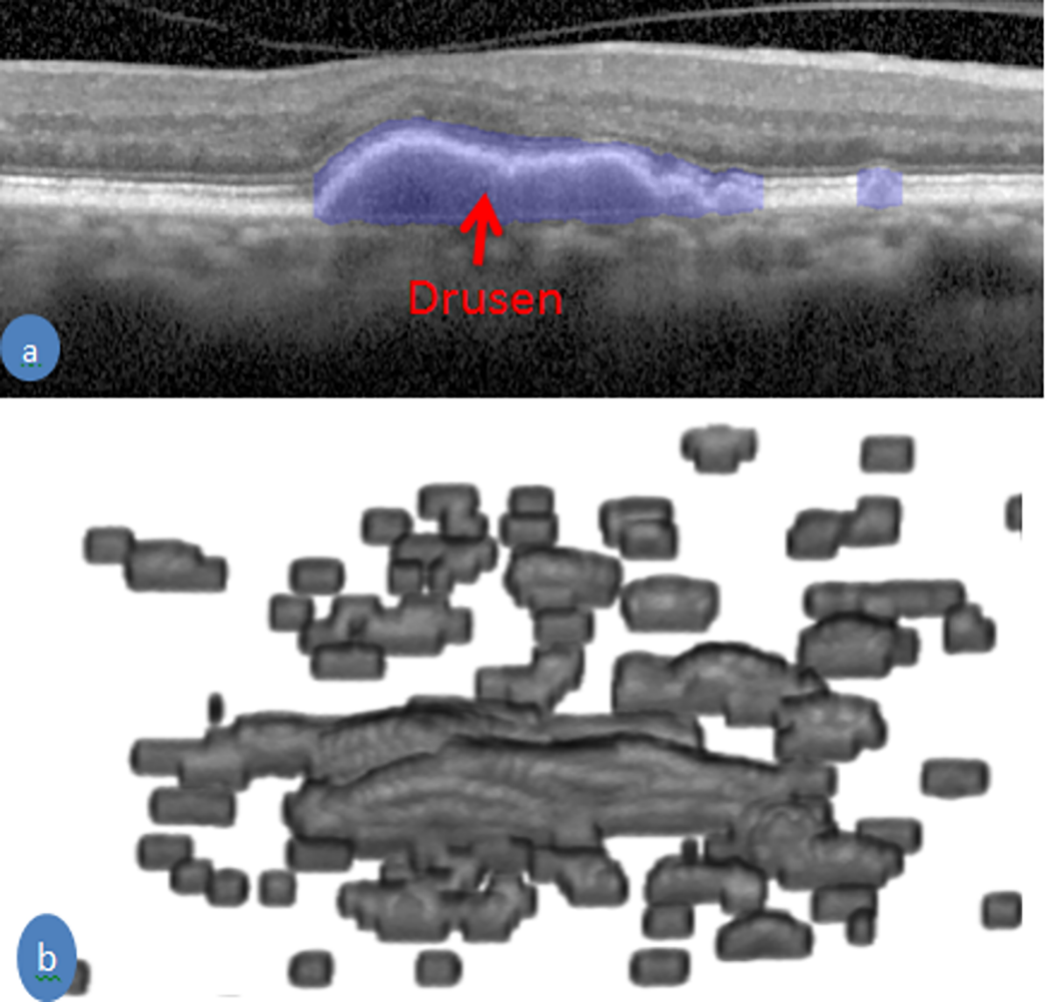
An example of drusen segmentation. (a) an SD-OCT B-scan with delineation of drusen by the blue color (b) Drusen in 3D view of an SD-OCT volume of an AMD patient.

The presence of drusen in the retinal SD-OCT images are a key risk factor for AMD patients [21, 22]. For this reason, we have chosen the volume of a druse as a feature for the classification method. They are characterised by the RPE layer detaching from the BM and intensities in the detached area are lower than the RPE layer as shown in Fig 1(g). This detachment changes the shape and position of the upper few layers all the way to ELM layer as shown in Fig 6(a). The thicknesses of ELM to BM layer are computed and a first order polynomial is used to fit the thickness with respect to horizontal position. If the value of the thickness deviates from the fitted value of the polynomial it is considered as potentially an area of drusen. If the intensity ratio between upper and lower few pixels in the potential drusen area is greater than 1.3 (set empirically), then it is considered to be drusen. The total number of pixels multiplied by the resolution of the image is considered as the volume of the drusen. Fig 6 shows an example of the automatic segmentation of the drusen area by our proposed method and a 3D view of drusen in the SD-OCT volume.

#### Feature 3: Curviness of the MZ-EZ boundary

**Fig 7:**
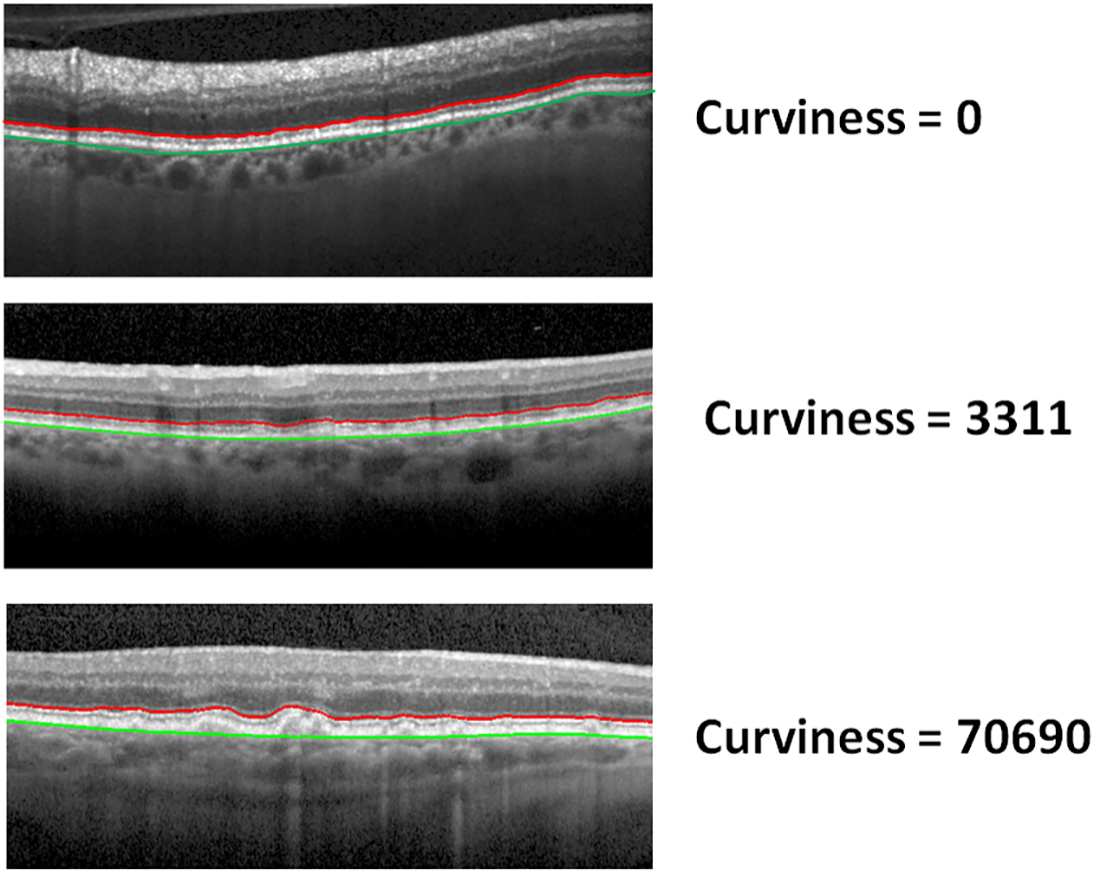
The curviness of different MZ-EZ boundaries. The curviness of different MZ-EZ boundaries (red colour) with a different curve using our proposed method. RBC boundary is in green colour.

The curviness (non-straightness) of the MZ-EZ boundary is an effect of RPE detachment and drusen for AMD. We have proposed a method to compute the curviness of a boundary that is shown in Algorithm 1. In step 1, the position of the RBC boundary is subtracted from the given boundary to normalise line the position. A constant value (*α* = 3) is used to penalise a position of the boundary as curviness. For example, if deficiency of the boundary from the first order polynomial value is more than *α*, we consider them as curvy and penalise otherwise they are not penalised. Another constant value (*δ* = 5) is used to find the peak of the boundary. For example, a position of the boundary is defined as peak, if it has at least *δ* difference between the local maxima and the local minima. A value is considered local maxima if that value is more than one of neighbours but not less than any of the neighbours. Similarly, a value is considered local minima if that value is less than one of the neighbours but not greater than any of the neighbours. This will ensure there is one local maxima between two local minima and vice versa. The nearest two local maxima and minima is removed if the difference between them is less than *δ*. This step is repeated for removing all neighbour local minima and maxima with difference less than *δ*. Fig 7 shows the curviness of different boundaries.

**Algorithm 1** Curviness of a given boundary

**Input:** The boundary of RBC (*B*_*RBC*_) and given (*B*_*Given*_).

**Output:** The value of curviness (C).

1: NL = *B*_*RBC*_—*B*_*Given*_.

2: Compute first order polynomial $P_{NL}^{1}$ using NL

3: Df = $|NL-P_{NL}^{1}|$

4: *C*_1_ = Σ_*iϵ*(*Df*>*α*))_*Df*_*i*_

5: LMM = *localMaxMin*(NL)

6: Repeat until there is at least one *minDiffNeigh*(LMM)<*δ*

7:   Remove Smallest Difference Pair in LMM

8: End Loop

9: *C*_2_ = Number of Local Maxima in LMM

10: C = *C*_1_ × *C*_2_.

*α* is the maximum deficiency value for not a curve. *localMaxMin*(NL) is a function that gives the local maxima and minima in the NL and stored into LMM; *minDiffNeigh*(LMM) is a function which gives minimum difference between neighbour minima and maxima.

#### Feature 4: Curviness of the OPL-ONL boundary

The curviness of the OPL-ONL boundary is an effect due to the Cyst and HIS exist for retinal disease DME. The curviness of this boundary is computed using Algorithm 1 with same constant values as MZ-EZ curviness (Feature 3).

#### Feature 5-10: Thickness parameters of the structures

**Fig 8:**
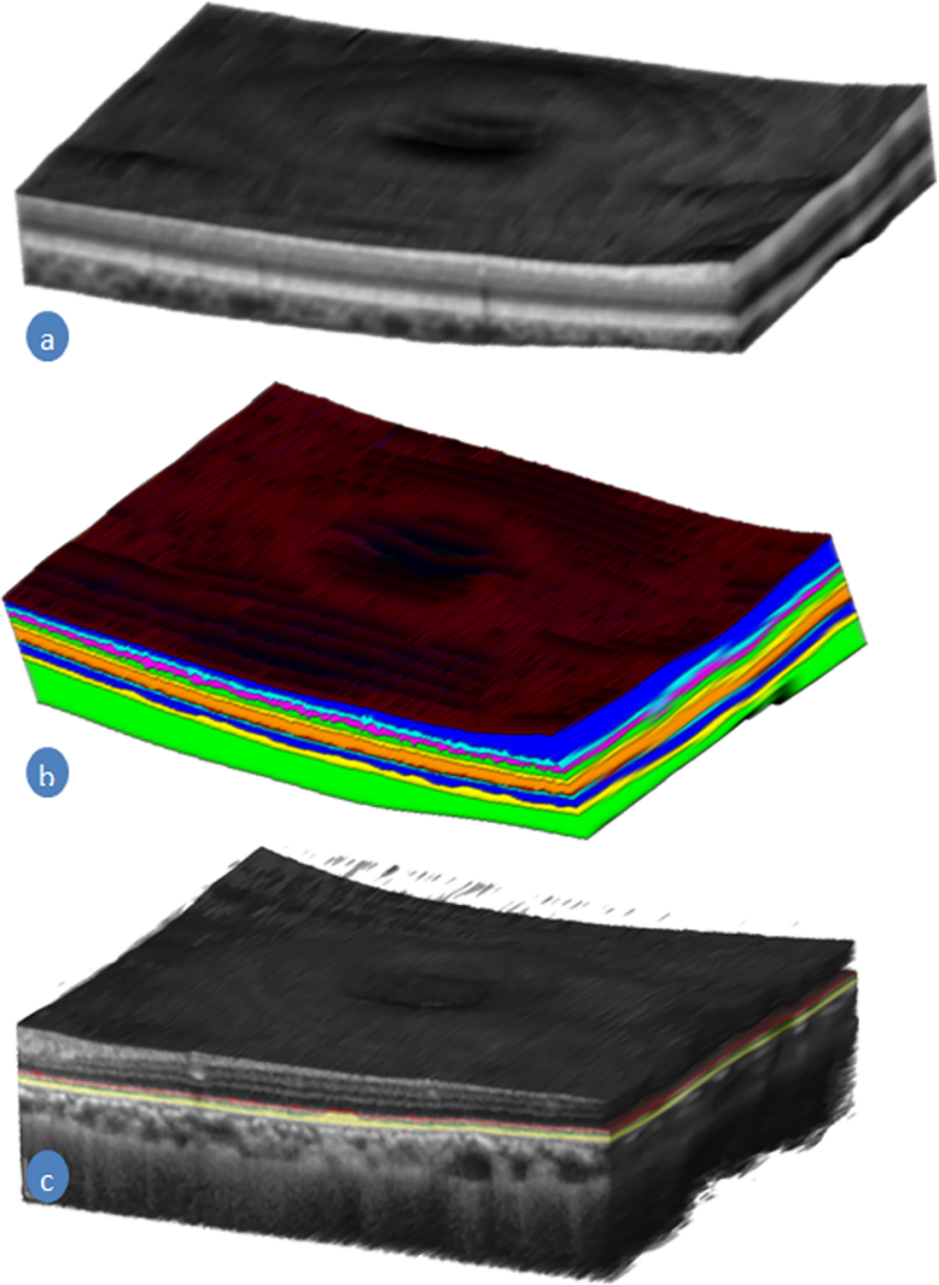
A volumetric image of the retina and the choroid. (a) A 3D render image of the retina with choroid constructed from an SD-OCT volume. (b) Segmented layers of the retina and choroid (c) The complex of the EZ, IZ, and RPE in a different colour in the gray-scale retinal SD-OCT image.

Six more features from three structures, Retina, Complex of EZ to RBC layers, and RPE layer are quantified as features shown in Fig 8. Two features from each of the structure are added to the feature list. The thicknesses of these structures of the retina have changed significantly due to the retinal diseases AMD and DME [15, 24]. For this reason, we have added mean and 70^th^ percentile of the thickness value of these structures as features for the classification method. The thickness of the structure is computed by the difference of the position of the enclosed boundaries such as the thickness of the retina is computed by ILM-RNFL and RBC boundaries. Similarly, the thickness of the complex of EZ to RBC layer is computed by ONL-EZ and RBC boundaries, and the thickness of the RPE layer is computed by IZ-RPE and RBC boundaries. The thickness is then smoothed for reducing the possible error in the segmentation by applying the Gaussian filter. Then these smooth thickness values are used for computing the features.

## Dataset and experiment setup

We obtained SD-OCT images from four sources: Duke University [17], Centre for Eye Research Australia (CERA), New York University (NYU) and Tian et al. [18]. The Duke University provided 45 subjects consisting of 15 normal, 15 AMD and 15 DME. CERA provided 20 Normal and 162 AMD subjects; NYU provided 14 normal subjects and Tian et al. provided 10 normal subjects. Among these four sources Duke University and Tian et al. images are publicly available. CERA images have 512 × 1024 × 49 voxels per subjects of SD-OCT volumes acquiring from Spectralis SD-OCT (Heidelberg Inc., Heidelberg, Germany). New York University images have 512 × 1024 × 49 voxels per subjects of EDI-OCT volumes acquiring from Spectralis SD-OCT (Heidelberg Inc., Heidelberg, Germany). The study was approved by the Institutional Review Board of The University of Melbourne and the Human Ethics Committee of the Royal Victorian Eye and Ear Hospital. Written informed consent conforming to the tenets of the Declaration of Helsinki was acquired from all participants. Using these four sources of images, we constructed seven datasets see Table 1. D1 is constructed with normal and DME subjects because Venhuizen et al. [19], Lemaitre et al. [2] and Sidibe et al. [20] report their methods performance on this partial data of Duke University. The datasets is constructed to show the performance of our proposed method in different sources of images and combine sources.

**Table 1:** Constructed datasets from four sources.

Dataset	Source	Normal	AMD	DME
D1	Duke University	15	0	15
D2	Duke University	15	15	15
D3	CERA	20	162	0
D4	Duke University + CERA	35	177	0
D5	Duke University + CERA	35	177	15
D6	Duke University + CERA + NYU + Tian	59	177	0
D7	Duke University + CERA + NYU + Tian	59	177	15

We are aiming to make public our collected dataset both CERA and NYU and currently, we are seeking ethics clearance from two medical organisations. Once approved, we will provide the download link and we expect do it before final publication of this paper. However, our used features of the subjects for the model are available in https://tinyurl.com/y7vy2lmr.

Several machine-learning algorithms are used to compare the accuracy of the classification model for two and three class classifications of eye patients. The machine-learning algorithm are Logistic Regression Model; Support Vector Machine with two kernel functions, Linear and Radial basis function; AdaBoost, Naive Bayes Model, Decision Tree with Regression and classification model; and Random Forest (RF); two class classification model categories the subjects into normal and diseased, while three class classification model categories into AMD, DME and normal. We have used Matlab default library function for each of the machine learning algorithms’ implementation [25]. We have performed k-fold cross validation with *k* = 15 for all machine learning algorithm on both datasets this way we make sure that each test fold has at least one instance of each case tested as in [17], In k-fold cross validation test, a given dataset is randomly divided into k parts (fold) where (k-1) folds of subjects are used for training the classification model and remaining one fold of subjects are used to test the model. The system is executed k times so that each fold of the subjects must be used once for testing the model. Since the given dataset is divided randomly into folds and the performance of the classification model depends on the training data, so the performance of the model varied in each iteration. For this reason, we have repeated the k-fold cross validation test 10 times and average accuracy (total number of successfully classified subjects divided by the total number of subjects) is defined as the model’s accuracy. The optimal parameters of the classification algorithms are selected for each fold using a portion of training data as validation data. Once the optimal parameters for the fold are chosen the model is learned using the whole training data and the model is tested using the test data. The optimal parameters chosen are number of trees for RF and Kernel scale for SVM.

## Result & discussion

The public data available by Srinivissan et al. [17] has 45 subjects and it contains three groups (Normal, DME and AMD) each containing 15 subjects. A part of this dataset (Normal and DME) is used by several researchers, and they are Lemaitre et al. [2] and Sidibe et al. [20]. These two researchers have also shown the performance of Venhuizen et al. [19] on the same dataset. So it is one of the best and accurate way to compare the performance of the methods on this partial dataset between our proposed method and the state -of-the-art methods. The comparison between state-of-the-art methods in terms of sensitivity, specificity, f1-score, accuracy and AUC value on this partial Duke dataset (D1) are shown in Table 2. Since Lemaitre et al. [2] and Sidibe et al. [20] have reported the performance of the classification model for two classes (Normal and DME) using sensitivity and specificity on the partial data of Duke dataset (only normal and DME patients), we have also followed the same approach for comparison purposes. We have reported the performance of Venhuizen et al. [19], Lemaitre et al. [2] and Sidibe et al. [20] on D1 dataset (only DME and Normal patients) from Sidibe et al. An Area Under the receiver operator characteristics Curve (AUC) value was not reported by these researchers which is why the corresponding cells in Table 2 contain “NA”. In the following paragraph, we explain the outcome of each method in details.

**Table 2:** Performance of four state-of-the-art and proposed methods on partial Duke dataset (D1) considering only normal and DME patients (because the Venhuizen et al., Lemaitre et al., and Sidibe et al. dataset only used these images).

Metric	Srinivisan et al.	Venhuizen et al.	Lemaitre et al.	Sidibe et al.	Proposed
Sensitivity	100	71.42	86.67	80	94.67
Specificity	86.67	68.75	100	100	100.00
f1-score	93.75	70.47	92.86	88.89	97.22
Accuracy	93.33%	70.0%	93.33%	90.00%	97.33%
AUC value	NA	NA	NA	NA	0.99

The sensitivity of Venhuizen et al., Lemaitre et al. and Sidibe et al. are less than 90, Srinivissan et al. achieve 100 and our proposed method achieve 94.67. On the other hand, Srinivissan achieve less than 90 for specificity while Lemaitre et al., Sidibe et al. and our proposed method achieves 100. Another important metric for the performance measurement is f1-score, where our proposed method achieves the highest value that is 97.22 and second highest value (93.75) is achieved by Srinivissan et al. Our proposed method achieve the highest value not only for f1-score but also for accuracy which is more than 97 where second highest value (93.33) is achieved by Srinivissan et al. and Lemaitre et al. We have also reported the AUC value which is not reported by these state-of-the-art methods. Liu et al. [13], Albarrak et al. [14] and Fraccaro et al. [15] use different dataset and not available for others, as a result, there is no way to compare the performance of methods against them. It might be possible to compare with them if we can imitate their implementations and apply on our dataset which is also not accurate always due to many choices of parameters. As a result, it is not possible to give a proper comparison with them. Although it is not a proper way to claim our method is better than their based on their reported performances of the methods, but regarding the AUC value, it is clear our method is better than them. Since all these reported methods except Fraccaro et al. use image intensity-based features for classification. We can see from the result that image intensity-based features are less accurate than retinal structure-based features. Also, Fraccaro et al. use druse and other pathologies quantity where we have used pathologies quantity as well as curviness of the layers which increase the accuracy. From these observations, it is clear that retinal layers’ changes are an important set of features for the classification problem.

We have performed more experiment on larger datasets (D1-D7, see Table 1) for our proposed classification method and reported in Table 3. Table 3 shows sensitivity, specificity, f1-score, accuracy and AUC for our proposed method in the means of mean (standard deviation) since we performed 15-fold cross validation test for ten times. We have used *k* = 15 for k-fold cross validation test using Random Forest classification method. It is the best way to evaluate the performance as each test fold contains one instance of each class for three class classification. The accuracy for our proposed method is better than Srinivisan et al. (the owner of the dataset D2) on dataset D2 where we have achieved an average accuracy 96.89% with standard deviation 2.15 for ten iteration of 15-fold cross validation tests while Srinivisan et al. achieved 95.56%. We have performed three-class classification test whenever there are three classes of subjects such as D2, D5 and D7 as reported in Table 3. We can observe that the classification accuracy decreases when only one kind of subjects increases or there is an imbalance in the number of subjects; for example the accuracy of three-class classification on dataset D5 and D7. However, our proposed method achieved high accuracy in all datasets in terms of each metric. In addition, we have achieved the AUC values for each case (for example, normal participants as positive class in three and two class classification method; AMD patients as positive class; DME as positive class; etc.) 0.99 with a standard deviation of 0.001.

**Table 3:** The metric in mean (standard deviation) of 10 iterations on 15-fold cross-validation test for the proposed classification model using Random Forest.

Dataset	# of class	Sensitivity	Specificity	f1-Score	Accuracy	AUC
D1	2	94.67(4.22)	100.00(0.00)	97.22(2.23)	97.33(2.11)	0.99(0.00)
D2	2	96.67(1.57)	100.00(0.00)	98.30(0.81)	97.78(1.05)	0.99(0.00)
	3				96.89(2.15)	0.99(0.00)
D3	2	99.14(0.32)	85.00(0.00)	98.65(0.16)	97.58(0.28)	0.99(0.00)
D4	2	98.64(0.40)	88.86(2.11)	98.23(0.33)	97.03(0.55)	0.99(0.00)
D5	2	98.28(0.25)	88.57(1.35)	98.10(0.18)	96.78(0.30)	0.99(0.00)
	3				94.63(0.54)	0.99(0.00)
D6	2	98.42(0.45)	97.29(0.88)	98.75(0.33)	98.14(0.50)	0.99(0.00)
D7	2	97.60(0.44)	96.10(0.82)	98.19(0.32)	97.25(0.48)	0.99(0.00)
	3				95.58(0.44)	0.99(0.00)

We have examined the classification model using several machine learning approaches. The accuracy in 15-fold cross validation test in the means of mean (standard deviation) for ten times in dataset D2 is reported in Table 4. Logistic regression model shows worst performance that demonstrates unsuitability as classification model for the patients based on the proposed features from the SD-OCT images. SVM with linear and RBF kernel functions show better result but not as good as AdaBoost, Naive Bayes Model, Decision Tree with Regression and classification model; and Random Forest (RF) based classification model. AdaBoost based classification model shows similar performance as SVM. Naive Bayes Model shows good accuracy for the binary classification where it shows 100% accuracy. Regression and Classification based decision tree also show good accuracy using our extracted features. Though classification based decision tree has better accuracy than regression based decision tree but the difference is small. RF shows accuracy more than 96.89% in all test with low standard deviation for each iteration of 15-fold cross validation test. Though, RF based classification model is not the best in accuracy in all cases, that is also true for others, but its consistency of accuracy in each test makes it superior for acceptance over others.

**Table 4:** The accuracy for different machine-learning algorithms for the classification model based on the proposed features on dataset D2.

# of class	LRM	SVM		AB	NBM	Decision Tree		RFT
		Linear	RBF			Reg.	Class.	
2	76.44 (3.18)	87.33 (1.50)	88.67 (1.26)	95.56 (1.48)	100.00 (0.00)	92.22 (2.40)	94.00 (1.50)	97.78 (1.05)
3	49.56 (2.58)	87.56 (1.55)	86.22 (1.75)	88.44 (3.89)	91.11 (1.81)	93.33 (0.00)	93.56 (0.70)	96.89 (2.15)

LRM: Logistic Regression Model; AB: AdaBoost; NBM: Naive Bayes Model; Reg.: Regression; Class.: Classification; RFT: Random Forest Tree

## Conclusion

In this paper, we have proposed a novel method of eye disease classification using automatically quantified hand-crafted clinical driven features of AMD, DME and normal participants using the Random Forest algorithm. We have also examined a number of machine learning algorithms, but RF performs best on accuracy compared to others in both dataset. The AUC value is also very high (0.99) with a small standard deviation 0.001 for the classification method. This high accuracy with several machine algorithms demonstrate the features extracted can model the disease. Moreover, we emphasise, this is the first method where completely automatic segmentation of the layers and extraction of pathologies are employed for classification of AMD, DME using SD-OCT images. The results show as predicted diseases are highly correlated with the layers’ thicknesses and pathologies. Though we have achieved higher accuracy than state-of-the-art methods, our method may have some limitation where further research may need to be performed. Our adapted retinal layer segmentation is one of the accurate methods among state-of-the-art methods; however, it can fail to detect the retinal layers in the presence of the extreme pathologies. So it is worth investing this situation and we believe sensitivity will increase and achieve better results. Another scope of future work after this analysis is testing the method on different levels of severity of diseases of subjects which will provide more reliability of the proposed methods. Also, the score of the automatic classifiers might be used as the severity level of the disease. Unfortunately, we currently do not have such data sets. In our future work, we plan to work with Ophthalmologists to build data sets containing severity levels for prediction. Other layers information such as ONL layer thickness, can also be considered as features for designing the classification model or the prediction model. Our proposed automated system can be employed for early detection of common eye diseases that do not have symptoms early in the disease progression state, allowing earlier intervention.
